# Maternal and neonatal outcomes in a cohort of pregnant women during conflict, Gaza Strip

**DOI:** 10.2471/BLT.25.293829

**Published:** 2026-03-30

**Authors:** Zeina Jamaluddine, Reham Jaffal, Sanaa Al Najjar, Mahmoud Al Kahlout, Tamam Abu Zeid, Hala Mughari, Ghada Al Jadba, Rami Habash, Bassam A Abu Hamad, Akihiro Seita, Oona Maeve Renee Campbell

**Affiliations:** a Faculty of Epidemiology and Population Health, London School of Hygiene and Tropical Medicine, Keppel Street, London, WC1E 7HT, England.; b United Nations Relief and Works Agency for Palestine Refugees in the Near East, Amman, Jordan.; c School of Public Health, Al-Quds University, Gaza City, occupied Palestinian territory, including east Jerusalem.

## Abstract

**Objective:**

To assess the impact of conflict on maternal and neonatal mortality and health-care utilization among pregnant refugee women in the Gaza Strip.

**Methods:**

The study involved data on pregnancy outcomes for 17 713 refugee women in the Gaza Strip who were pregnant on 8 October 2023 and received antenatal care from the United Nations Relief and Works Agency for Palestine Refugees in the Near East (UNRWA). We obtained data from UNRWA’s electronic health records, an UNRWA phone survey and the Palestinian Ministry of Health’s lists of deaths for October 2023 to June 2024.

**Findings:**

Overall, 168 maternal deaths were identified, yielding a pregnancy-related mortality ratio of 948 per 100 000 pregnancies compared to a maternal mortality ratio of 30 per 100 000 live births before the conflict. The pattern of age-specific, pregnancy-related deaths suggested that most were trauma-related. The stillbirth rate among women with known pregnancy outcomes was 10.3 per 1000 births compared with 6.0 per 1000 before the conflict, and the neonatal mortality rate was 9.4 per 1000 live births compared with 6.6 per 1000. The preterm birth rate was also higher, at 13.6% versus 9.7% before the conflict. Although most deliveries (97.3%) remained facility-based, there was a shift from government to nongovernmental organization facilities.

**Conclusion:**

The conflict in the Gaza Strip was associated with substantial increases in maternal and neonatal mortality and preterm births. Utilization of health services remained high, but delivery locations shifted to lower-level facilities and hospital stays shortened, raising concerns about the quality of care.

## Résumé

**Objectif:**

Évaluer l’impact du conflit sur la mortalité maternelle et néonatale ainsi que sur le recours aux soins de santé chez les femmes enceintes réfugiées dans la bande de Gaza.

**Méthodes:**

L’étude se base sur des données relatives à l’issue de la grossesse de 17 713 femmes réfugiées de la bande de Gaza qui étaient enceintes au 8 octobre 2023 et qui ont bénéficié de soins prénatals dispensés par l’Office de secours et de travaux des Nations Unies pour les réfugiés de Palestine dans le Proche-Orient (UNRWA). Ces données proviennent des dossiers médicaux électroniques de l’UNRWA, d’une enquête téléphonique menée par l’UNRWA et des listes de décès du ministère palestinien de la Santé pour la période d’octobre 2023 à juin 2024.

**Résultats:**

En tout, 168 décès maternels ont été recensés, ce qui correspond à un taux de mortalité de 948 pour 100 000 grossesses, contre un taux de 30 pour 100 000 naissances vivantes avant le conflit. La répartition par âge des décès liés à la grossesse suggère que la plupart étaient dus à des traumatismes. Le taux de mortinaissance chez les femmes dont l’issue de la grossesse était connue s’élevait à 10,3 pour 1 000 naissances, contre 6,0 pour 1 000 avant le conflit, et le taux de mortalité néonatale était de 9,4 pour 1 000 naissances vivantes, contre 6,6 pour 1 000. Le taux de naissances prématurées était également plus élevé, s’établissant à 13,6% contre 9,7% avant le conflit. Bien que la plupart des accouchements (97,3%) aient encore lieu dans un établissement médical, on a observé un transfert depuis des établissements publics vers ceux d’organisations non gouvernementales.

**Conclusion:**

Le conflit dans la bande de Gaza était associé à des augmentations substantielles de la mortalité maternelle et néonatale ainsi que des naissances prématurées. Le recours aux services de santé est resté élevé, mais les lieux d’accouchement se sont déplacés vers des établissements de niveau inférieur et la durée des séjours à l’hôpital s’est raccourcie, ce qui soulève des inquiétudes quant à la qualité des soins.

## Resumen

**Objetivo:**

Evaluar el impacto del conflicto en la mortalidad materna y neonatal y en la utilización de los servicios sanitarios entre mujeres refugiadas embarazadas en la Franja de Gaza.

**Métodos:**

El estudio incluyó datos sobre los resultados del embarazo de 17 713 mujeres refugiadas en la Franja de Gaza que estaban embarazadas el 8 de octubre de 2023 y recibían atención prenatal de la Agencia de Obras Públicas y Socorro de las Naciones Unidas para los Refugiados de Palestina en el Cercano Oriente (UNRWA). Los datos se obtuvieron de los registros electrónicos de salud de la UNRWA, de una encuesta telefónica de la UNRWA y de las listas de defunciones del Ministerio de Salud de Palestina correspondientes al periodo de octubre de 2023 a junio de 2024.

**Resultados:**

En total, se identificaron 168 muertes maternas, lo que supone una razón de mortalidad relacionada con el embarazo de 948 por 100 000 embarazos, en comparación con una razón de mortalidad materna de 30 por 100 000 nacidos vivos antes del conflicto. El patrón de muertes relacionadas con el embarazo específicas por edad sugiere que la mayoría estaban relacionadas con traumatismos. La tasa de mortinatalidad entre las mujeres con resultados del embarazo conocidos fue de 10,3 por 1000 nacimientos, en comparación con 6,0 por 1000 antes del conflicto, y la tasa de mortalidad neonatal fue de 9,4 por 1000 nacidos vivos, frente a 6,6 por 1000. La tasa de nacimientos prematuros también fue mayor, con un 13,6% frente al 9,7% antes del conflicto. Aunque la mayoría de los partos (97,3%) siguieron teniendo lugar en centros sanitarios, se observó un desplazamiento desde centros gubernamentales hacia centros gestionados por organizaciones no gubernamentales.

**Conclusión:**

El conflicto en la Franja de Gaza se asoció con aumentos sustanciales de la mortalidad materna y neonatal y de los nacimientos prematuros. La utilización de los servicios sanitarios se mantuvo elevada, pero los lugares de parto se desplazaron hacia centros de menor nivel y la duración de las estancias hospitalarias se redujo, lo que suscita preocupaciones sobre la calidad de la atención.

## ملخص

**الغرض:**

تقييم أثر النزاع على وفيات الأمهات والمواليد، وعلى الاستفادة من الرعاية الصحية بين اللاجئات الحوامل في قطاع غزة.

**الطريقة:**

شملت الدراسة بيانات عن نتائج الحمل لعدد 17713 لاجئة في قطاع غزة، اللاتي كنّ حوامل في 8 أكتوبر/تشرين الأول 2023، وتلقين رعاية ما قبل الولادة من وكالة الأمم المتحدة لإغاثة وتشغيل اللاجئين (الأونروا) الفلسطينيين في الشرق الأدنى. تم الحصول على البيانات من السجلات الصحية الإلكترونية للأونروا، واستطلاع رأي عبر الهاتف أجرته الوكالة، وقوائم الوفيات الصادرة عن وزارة الصحة الفلسطينية للفترة من أكتوبر/تشرين الأول 2023 إلى يونيو/حزيران 2024.

**النتائج:**

تم رصد 168 حالة وفاة للأمهات، ما يمثل نسبة وفيات مرتبطة بالحمل بلغت 948 حالة لكل 100000 حمل، مقارنةً بنسبة وفيات للأمهات بلغت 30 حالة لكل 100000 مولود حي قبل النزاع. أظهر نمط الوفيات المرتبطة بالحمل، بحسب الفئة العمرية، أن معظمها ناجم عن إصابات. وبلغ معدل ولادة الأجنة الميتة بين النساء اللواتي عُرفت نتائج حملهن 10.3 لكل 1000 حالة ولادة، مقارنةً بـ 6.0 لكل 1000 قبل النزاع، بينما بلغ معدل وفيات المواليد 9.4 لكل 1000 مولود حي، مقارنةً بـ 6.6 لكل 1000. كما ارتفع معدل الولادة المبكرة إلى %13.6 مقابل %9.7 قبل النزاع. ورغم أن معظم الولادات (%97.3) تمت في المرافق الصحية، إلا أن هناك تحولاً من المرافق الحكومية إلى مرافق المنظمات غير الحكومية.

**الاستنتاج:**

ارتبط النزاع في قطاع غزة بارتفاعات ذات صلة ملموسة في وفيات الأمهات والمواليد والولادات المبكرة. وظلت الاستفادة من الخدمات الصحية مرتفعة، لكن أماكن الولادة تحولت إلى مرافق أقل مستوى، وانخفضت مدة الإقامة في المستشفيات، مما أثار مخاوف بشأن الجودة النوعية للرعاية المقدمة.

## 摘要

**目的:**

旨在评估冲突对加沙地带妊娠期难民妇女中的孕产妇和新生儿死亡率以及医疗保健服务使用率的影响。

**方法:**

该项研究采用加沙地带 17,713 名在 2023 年 10 月 8 日仍处于妊娠期并在联合国近东巴勒斯坦难民救济和工程处 (UNRWA) 接受过产前护理的难民妇女的妊娠结局相关数据。这些数据来源于 UNRWA 的电子健康档案、UNRWA 的一项电话调查以及巴勒斯坦卫生部公布的 2023 年 10 月至 2024 年 6 月期间的死亡名单。

**结果:**

我们发现孕产妇死亡人数总共为 168 例，从而得出冲突期间每 10 万例妊娠的妊娠相关死亡率为 948 例，相比之下，冲突发生前每 10 万例活产的孕产妇死亡率仅为 30 例。年龄别妊娠相关死亡人数分布特征表明，大多数死亡都与创伤有关。在已知妊娠结局的妇女中，冲突期间每 1000 例分娩的死产率为 10.3 例，相比之下，冲突发生前每 1000 例分娩的死产率仅为 6.0 例；冲突期间每 1000 例活产的新生儿死亡率为 9.4 例，相比之下，冲突发生前每 1000 例活产的新生儿死亡率仅为 6.6 例。冲突期间的早产率 (13.6%) 也高于冲突发生前 (9.7%)。尽管大多数孕妇 (97.3%) 仍然选择在医疗机构进行分娩，但已逐步从政府组织运营的医疗机构转向非政府组织运营的医疗机构。

**结论:**

加沙地带的冲突导致孕产妇和新生儿死亡率以及早产率大幅上升。尽管保健服务的使用率始终处于较高水平，但分娩地点已逐步转向了级别较低的医疗机构，且住院时间也有所缩短，这引发了人们对护理质量的担忧。

## Резюме

**Цель:**

Оценить влияние войны на смертность среди матерей и новорожденных, а также на частоту обращения за медицинской помощью среди беременных беженок в секторе Газа.

**Методы:**

В исследование были включены данные по исходам беременности у 17 713 беженок в секторе Газа, которые были беременны по состоянию на 8 октября 2023 года и получали дородовый уход со стороны Ближневосточного агентства Организации Объединенных Наций для помощи палестинским беженцам и организации работ (БАПОР). Данные были получены из электронных медицинских карт БАПОР, телефонного опроса, проведенного БАПОР, а также из списков умерших за период с октября 2023 г. по июнь 2024 г., полученных от Министерства здравоохранения Палестины.

**Результаты:**

В общей сложности было выявлено 168 случаев смерти матерей, что соответствует коэффициенту смертности в связи с беременностью на уровне 948 случаев на 100 000 беременностей по сравнению с коэффициентом 30 смертей на 100 000 живорождений до начала войны. Возрастная структура случаев смерти, связанных с беременностью, позволяет предположить, что большинство из них были вызваны травмами. Коэффициент мертворождаемости среди женщин с известными исходами беременности составил 10,3 на 1000 рождений по сравнению с 6,0 на 1000 рождений до начала войны, а коэффициент смертности среди новорожденных составил 9,4 на 1000 живорождений по сравнению с 6,6 на 1000 живорождений. Частота преждевременных родов также повысилась с довоенных 9,7 до 13,6%. Хотя большая часть родов (97,3%) происходили в медицинских учреждениях, наблюдался отход от использования государственных клиник в пользу неправительственных учреждений.

**Вывод:**

Война в секторе Газа ассоциировалась со значительным ростом смертности среди матерей и новорожденных, а также с повышением частоты преждевременных родов. Показатели обращения за медицинской помощью оставались высокими, но роды чаще происходили в учреждениях более низкой категории, сроки пребывания в больнице сократились, что вызывает сомнения в качестве медицинского обслуживания.

## Introduction

In the Gaza Strip, the maternal mortality ratio between 2018 and 2022 averaged 30 per 100 000 live births,^1^ which was substantially lower than the global average of 223 per 100 000 in 2020.^2^ According to the Palestinian Ministry of Health, the neonatal mortality rate was 6.6 per 1000 live births in 2022,^1^ which again compared favourably to the 17.3 per 1000 reported globally in 2024;^3^ the stillbirth rate was 6.0 per 1000 births,^1^ which was also lower than the global rate of 13.9 per 1000.^3^ In addition, high proportions of antenatal care (100%), skilled birth attendance (100%) and facility-based delivery (99.4%) confirmed that the health-care system was robust.^4^ As of 2020, the total fertility rate was also high, at 3.9 births per woman.^4^

In 2017, around 48% of women aged 15 to 49 years in the Gaza Strip were original inhabitants (i.e. non-refugees), whereas the remaining 52% were descendants of individuals displaced from their homes in 1948 (i.e. refugees).^5^ The United Nations Relief and Works Agency for Palestine Refugees in the Near East (UNRWA) provides free primary health-care services to Palestinian refugees through 22 health centres in the Gaza Strip, including antenatal care and partial support for childbirth.^6^ In 2010, UNRWA developed an electronic health record (e-health) system that generates comprehensive maternal and neonatal health data. The system includes the registration of pregnancies during antenatal care.^7^

The conflict in the Gaza Strip which started on 7 October 2023 severely disrupted health-care services, including UNRWA’s. Health infrastructure was extensively damaged by the Israeli armed forces and many health personnel were killed.^8^^–^^11^ By January 2025, emergency obstetric and newborn care were available only in seven of 18 partially functioning hospitals, in four of 11 field hospitals and in one community health centre.^12^ Access to life-saving emergency obstetric and newborn care was drastically limited and there were reports of caesarean sections being conducted without anaesthesia or electricity.^13^ The entire population of the Gaza Strip had an insecure food supply and limited access to water.^14^ In 2021, over half of pregnant women in the Gaza Strip were anaemic,^15^ which increased the risk of preterm birth and intrauterine fetal growth restriction. Both these conditions had already been made more likely by malnutrition. In addition, infectious disease outbreaks were occurring, which in turn also affected underlying health and nutritional status.^16^

The aims of our study were to derive indicators of maternal and neonatal mortality and of health service use in the Gaza Strip after the outbreak of the conflict and to compare these indicator values to equivalents before the conflict started. We analysed data from UNRWA’s e-health records, which were supplemented by a phone survey conducted by UNRWA and by data on deaths recorded by the Palestinian Ministry of Health.

## Methods

Our study employed a closed cohort design with a prospective follow-up. All refugee women residing in the Gaza Strip who were recorded as pregnant in UNRWA’s e-health records on 8 October 2023 were eligible for inclusion. However, our study cohort comprised only women who had either registered their pregnancy through UNRWA’s antenatal care service or notified UNRWA of their pregnancy before 8 October 2023. These women were expected to have given birth by 30 June 2024.

### Data sources

#### UNRWA phone survey

Some women in the cohort had accessed primary health-care centres with functioning computers and had had their pregnancy outcomes recorded on UNRWA’s e-health system. Eligible women without an e-health record of their pregnancy outcome were followed up by phone to obtain data that supplemented the information extracted from their e-health records (details are available from the online repository).^17^ Nurses working with UNRWA in the West Bank volunteered to make the calls. The nurses underwent standardized training, which included protocols for sensitive questioning and data verification procedures, and were debriefed after calling. Up to three attempts were made to contact each woman between May and September 2024. Responses to the phone survey were cross-referenced with the available e-health records for consistency.

#### Palestinian Ministry of Health’s lists of deaths

On 23 October 2023, the Palestinian Ministry of Health began issuing lists of deaths that had occurred since 7 October 2023. List number five, dated 30 June 2024, covered the period during which women in the study cohort would have been expected to give birth and to complete 42 days postpartum. This list reported 28 185 deaths among the entire population of the Gaza Strip, including UNRWA refugees, and recorded each individual’s Palestinian registration identification (ID) number, name, sex, age at death and month of death. However, there were indications that deaths may have been underreported.^18^

We used exact (deterministic) matching to link the Palestinian Ministry of Health ’s lists of deaths with UNRWA records using Palestinian ID numbers. If the death of a woman in the study cohort was recorded in the UNRWA system but there was no record linked to her ID number in the Palestinian Ministry of Health lists, the lists were searched for her name, allowing for spelling variations in both her first name and surname.

#### Maternal and neonatal indicators before the conflict

Data on maternal and neonatal health indicators for the Gaza Strip for the period before 7 October 2023 were extracted from UNRWA’s e-health records for 2020,^7^ from Gaza health ministry reports for 2022,^1^ and from the 2019 to 2020 Palestine multiple indicator cluster survey,^4^ which was part of a household survey programme developed by the United Nations Children’s Fund. We re-analysed cluster survey data to generate indicators for the Gaza Strip alone, rather than for all of the occupied Palestinian territory, including east Jerusalem.

### Data analysis

We used data on women whose pregnancy outcome was known because they responded to the phone survey or their e-health records were available to produce values for the following indicators: (i) the pregnancy-related mortality ratio; (ii) the stillbirth rate; (iii) the neonatal mortality rate; (iv) the preterm birth rate; (v) the proportion of births in a health facility; (vi) the proportion of births with a skilled birth attendant; (vii) the length of stay in health facilities; and (viii) the caesarean section rate (Box 1). In addition, we derived a pregnancy-related mortality ratio for all women using data from the phone survey, e-health records and the Palestinian Ministry of Health ’s lists of deaths. A pregnancy-related death was defined as a death that resulted from: (i) a direct maternal cause related to the pregnancy or delivery; (ii) an indirect maternal cause due to the pregnancy exacerbating a pre-existing condition; or (iii) another cause, such as trauma.

Box 1 **Indicator definitions, study of maternal and neonatal outcomes in pregnant women, Gaza Strip, 2023–2024**

***Pregnancy-related mortality ratio***

The number of all deaths of women during pregnancy and childbirth and within 42 days of termination of the pregnancy, regardless of cause, per 100 000 pregnancies

***Stillbirth rate***

The number of fetal deaths at 28 weeks’ gestation or later (i.e. stillbirths) per 1000 births

***Neonatal mortality rate***

The number of neonates who died within the first 28 days of life per 1000 live births

***Preterm birth rate***

The percentage of live births that occurred before 37 weeks’ gestation

***Location of delivery distribution***

The percentage of stillbirths and live births delivered in a specific location (e.g. a hospital or home)

***Skilled birth attendance prevalence***

Percentage of stillbirths and live births attended by a skilled birth attendant (e.g. a midwife or doctor)

***Length of stay in a health facility***

Average duration (in hours) of mothers’ stays in a facility after delivery, by mode of delivery (i.e. caesarean section or vaginally)

***Caesarean section rate***

Percentage of stillbirths and live births delivered by caesarean section

As the Palestinian Ministry of Health listed deaths by the month of death rather than the date, for our analysis we assumed that deaths occurred mid-month (i.e. on the 15th). In addition, we conducted a sensitivity analysis in which we assumed that all deaths occurred on either the first or last day of the month, respectively. We used UNRWA’s expected delivery dates to determine whether a death occurred within 42 days of the expected end of pregnancy (that is, it was pregnancy-related). This approach, used in Reproductive Age Mortality Studies (RAMOS),^19^ identifies deceased women of reproductive age and ascertains their pregnancy or postpartum status at the time of death in a variety of ways, such as: (i) through links to pregnancy records (as we did); (ii) through links to birth records; (iii) by interviewing the families of the deceased women; or (iv) by using multiple information sources.

We performed separate analyses of deaths of women: (i) who responded to the phone survey or whose pregnancy outcomes were reported in e-health records; and (ii) women who were unreachable by phone and whose pregnancy outcomes were not reported in e-health records but who were included in the Palestinian Ministry of Health’s lists of deaths. First, we calculated mortality indicators for women with known pregnancy outcomes and noted the proportion of their deaths included on the Palestinian Ministry of Health’s lists. Second, we added pregnancy-related deaths among women with unknown pregnancy outcomes using the RAMOS linkage approach. Third, we assumed that the degree of underreporting by the Palestinian Ministry of Health was the same for women with unknown pregnancy outcomes as for those with known pregnancy outcomes. We did not perform any poststratification analysis or use weighting to account for differential responses by subregion.

We were able to calculate stillbirth and neonatal mortality rates only for women with known pregnancy outcomes. As stillbirths were not reported on the Palestinian Ministry of Health’s lists of deaths, it was not possible to identify stillbirths among women with unknown pregnancy outcomes. Similarly, these lists did not distinguish between neonatal and infant deaths. Instead, deaths were reported for infants younger than 1 year. Moreover, the phone survey did not ask for newborn ID numbers or names, so neonatal deaths could not be linked to Palestinian Ministry of Health lists. Nevertheless, we estimated stillbirth and neonatal mortality rates for the entire study cohort by assuming that the relationship between rates in women with unknown pregnancy outcomes and rates in women with known pregnancy outcomes was the same as the corresponding relationship observed for pregnancy-related deaths. All analyses were performed using Stata v.18 (StataCorp LLC, College Station, United States of America).

Approval to use the data was obtained from the ethics committee of the London School of Hygiene and Tropical Medicine (LSHTM Ethics Ref: 31273) and UNRWA’s research review board.

## Results

**Fig. 1:**
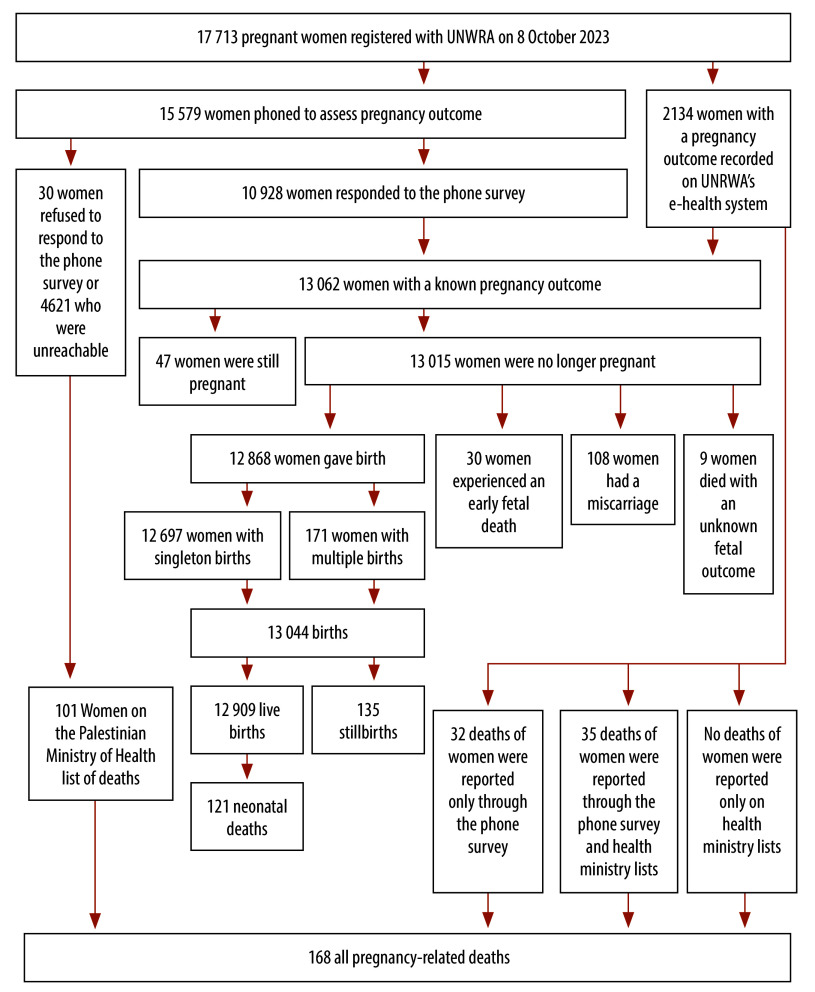
Flowchart, study of maternal and neonatal outcomes in pregnant women, Gaza Strip, 2023–2024

In total, data for 18 038 women were extracted from UNRWA records. As 325 women were ineligible because they had given birth before 8 October 2023 (and before their expected delivery dates), the study analysis involved the records of a cohort of 17 713 women who were pregnant on 8 October 2023 and who received antenatal care from UNRWA (Fig. 1). The size of the cohort was consistent with the number of women who attended UNRWA antenatal care services on 7 October 2020, which was 17 351,^17^ and was consistent with the expected pattern of age-specific fertility (details are available from the online repository).^17^

UNWRA: United Nations Relief and Works Agency for Palestine Refugees in the Near East.

Notes. Information on pregnancy outcomes was obtained by phone survey and from e-health records established by UNRWA. Among women who died with an unknown fetal outcome, five had a fetus in utero and four had an unknown fetal status. Multiple births included 332 twins and 15 triplets.

For the 17 713 women pregnant on 8 October 2023, information on pregnancy outcomes was obtained from UNRWA’s e-health system for 2134 (12.0%) and was sought through the phone survey for 15 579 (88.0%; Fig. 1).^17^ However, of the 15 579 followed up by phone, 30 (0.2%) refused to respond and 4621 (29.7%) were unreachable. In addition, 47 women were still pregnant. There was no significant difference in maternal age between women whose pregnancy outcomes were known and those whose outcomes were not known. However, the proportion of women who responded to calls or whose pregnancy outcomes were recorded in the e-health system varied greatly by the registered location of the clinic before the conflict. Overall, pregnancy outcomes were known for 13 015 women from either phone responses or e-health records: 12 868 had given birth after 28 weeks’ gestation; 108 had had miscarriages; 30 had experienced an early fetal death; and nine had died with their pregnancy outcomes not recorded (Fig. 1). The 12 868 pregnancies resulted in a total of 13 044 live births and stillbirths, including 332 twins and 15 triplets. Details of a sensitivity and specificity analysis of linkage to Palestinian Ministry of Health lists of deaths are available from the online repository.^17^

### Maternal and fetal deaths

No pregnancy-related deaths were recorded on UNRWA’s e-health system. However, the phone survey identified 67 pregnancy-related deaths among the 10 928 successfully contacted women: 35 were also recorded in the Palestinian Ministry of Health lists of deaths and 32 were unique to the phone survey. Among women contacted by phone, there were no deaths on Palestinian Ministry of Health lists that were missed by the phone survey. Nevertheless, 101 deaths on Palestinian Ministry of Health lists were linked to the 4651 women who were either unreachable by phone or refused to respond. Thus, a total of 168 pregnancy-related deaths were identified by combining data from Palestinian Ministry of Health ’s lists and the phone survey. In the sensitivity analysis, which regarded the day of death in Palestinian Ministry of Health lists as being the first or last day of the month rather than the 15th and which assumed that the end of the puerperium occurred 42 days after the expected delivery date, the estimated number of pregnancy-related deaths in the study cohort was 166 and 171 for these two days, respectively.

**Fig. 2:**
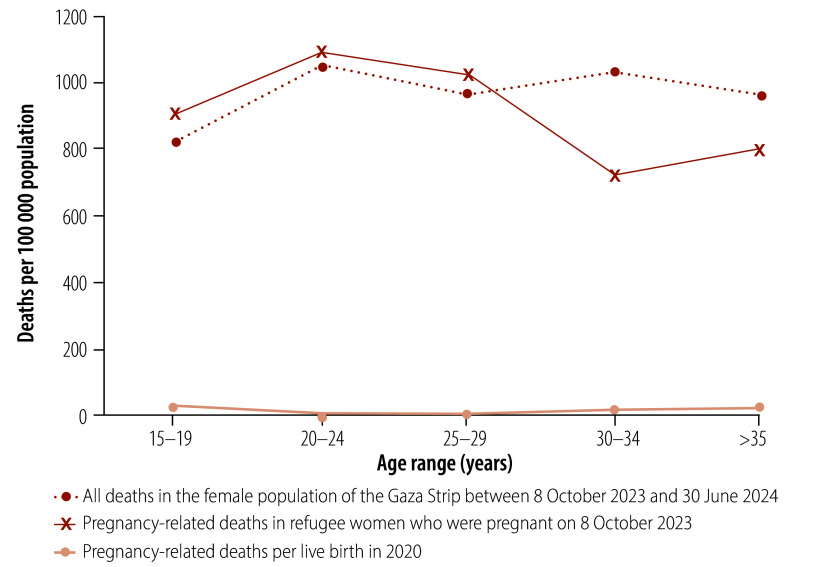
Deaths of women in the Gaza Strip before and after the start of the conflict, study of maternal and neonatal outcomes in pregnant women, Gaza Strip, 2023–2024

Notes. Female death rates between 8 October 2023 and 30 June 2024 were derived from lists of deaths issued by the Palestinian Ministry of Health.^18^ Data on pregnancy-related deaths in women registered as pregnant on 8 October 2023 were derived in the current study. We derived data on maternal mortality ratios for 2020 from United Nations Population Fund data for that year.^20^

Using a denominator of 17 713 pregnancies, the overall pregnancy-related mortality ratio derived from the combined data was 948 per 100 000 pregnancies (Table 1). Table 1 also reports 95% confidence intervals (95% CIs) and the implications of uncertainty in the assumed dates of death in Palestinian Ministry of Health lists. Assuming that Palestinian Ministry of Health data underestimated the number of deaths among women with unknown pregnancy outcomes to the same extent as among women with known pregnancy outcomes, the total number of pregnancy-related deaths was estimated to be 260 (193 among women with unknown pregnancy outcomes and 67 among women with known pregnancy outcomes), which corresponds to a pregnancy-related mortality ratio of 1468 per 100 000 pregnancies (Table 1). We also calculated age-specific, pregnancy-related mortality rates (per 100 000 women) using projected population data from the United Nations Population Fund for May 2023. Age-specific, maternal mortality ratios (i.e. per 100 000 live births) for 2020 were derived using Palestinian Ministry of Health and multiple indicator cluster survey data (Fig. 2).^4^ Age-specific mortality rates for all women in the Gaza Strip between 8 October and 30 June 2024 are also shown in Fig. 2.^20^^–^^22^

**Table 1:** Maternal and fetal deaths, study of maternal and neonatal outcomes in pregnant women, Gaza Strip, 2023–2024

Indicator and study population	No. women in study population^a^	Indicator value
**Pregnancy-related mortality ratio**		
All pregnant women,^b^ assuming deaths occurred on the 15^th^ of each month (no correction for Palestinian Ministry of Health underreporting of deaths)^c^	17 713	948 (95% CI: 805–1092) per 100 000 pregnancies
All pregnant women,^b^ assuming deaths occurred at the start or end of each month, respectively, rather than mid-month (no correction for Palestinian Ministry of Health underreporting of deaths)^d^	17 713	937–965 per 100 000 pregnancies
All pregnant women,^b^ with a correction for Palestinian Ministry of Health underreporting of deaths for women who refused to respond to, or were unreachable by, the phone survey	17 713	1468 (95% CI: 1291–1645) per 100 000 pregnancies
Women whose pregnancy outcome was known^e^	13 062	513 (95% CI: 390–635) per 100 000 pregnancies
Women who refused to respond to, or were unreachable by, the phone survey (no correction for Palestinian Ministry of Health underreporting of deaths)	4 651	2172 (95% CI: 1753–2591) per 100 000 pregnancies
Women who refused to respond to, or were unreachable by, the phone survey, with a correction for Palestinian Ministry of Health underreporting of deaths	4 651	4150 (95% CI: 3577–4723) per 100 000 pregnancies
**Stillbirth rate**		
Women whose pregnancy outcome was known and who had a livebirth or stillbirth	13 044^f^	10.3 (95% CI: 8.6–12.1) per 1000 births
**Neonatal mortality rate**		
Women whose pregnancy outcome was known and who had a live birth	12 909^g^	9.4 (95% CI: 7.7–11.0) per 1000 live births
**Preterm delivery rate**		
Women whose pregnancy outcome was known and who had a livebirth with a known gestational age^e^	12 127^h^	13.6% (95% CI: 13.0–14.2) of live births

CI: confidence interval; UNRWA: United Nations Relief and Works Agency for Palestine Refugees in the Near East.

^a^ The numbers listed are for women who were pregnant on 8 October 2023, unless otherwise indicated.

^b^ All refugee women residing in the Gaza Strip who were recorded as pregnant on 8 October 2023 in e-health records established by UNRWA.

^c^ The health ministry listed deaths by month. For the main analysis, we assumed all deaths occurred on 15th of each month.

^d^ The health ministry listed deaths by month. In a sensitivity analysis, we assumed that all deaths occurred on the first or last day of the month, respectively, rather than on the 15th, as assumed in the main analysis.

^e^ Information about pregnancy outcomes was obtained through an UNRWA phone survey or from UNRWA e-health records.

^f^ The number shown is for all births.

^g^ The number shown is for live births.

^h^ The number shown is for live births to women whose gestational age was known. Data on gestational age were missing for 782 live births.

An UNRWA social autopsy, which was conducted in 2024 separately from our phone study, identified 21 maternal deaths among pregnant refugee women that were not related to trauma: nine from postpartum haemorrhage, three from sepsis, three from other infections, two with unclear causes, and one each of renal failure, Ogilvie syndrome, pulmonary embolism and eclampsia (details are available from the data repository).^17^

Of the 13 044 births for which information was available through the phone survey or the e-health system, 135 were stillbirths, which yielded a stillbirth rate of 10.3 per 1000 births (Table 1). Among the 12 909 live births, there were 121 neonatal deaths, which yielded a neonatal death rate of 9.4 per 1000 live births (Table 1). The ratio of the estimated number of pregnancy-related deaths after correction for Palestinian Ministry of Health underreporting (i.e. 260) to the number of pregnancy-related deaths among women with known pregnancy outcomes (i.e. 67) was 2.88. If we assume the same ratio applies to stillbirths and neonatal deaths, the stillbirth and neonatal mortality rates would be as high as 29.7 per 1000 births and 27.0 per 1000 live births, respectively.

Among the 67 women who were reported to have died on the phone survey, four had an unknown pregnancy outcome, five died with a fetus in utero but with an unknown gestation period, five died before 22 weeks’ gestation (i.e. died with a non-viable fetus), five died between 22 and 27 weeks’ gestation (i.e. early fetal death), 21 died with a stillbirth (i.e. at more than 28 weeks’ gestation) and 27 had a live birth (12 of these neonates died in the neonatal period). The resulting neonatal mortality rate in the 27 mothers who died but had a live birth was 444 per 1000 live births. Among the 63 women who died and whose pregnancy outcomes were known, only 15 neonates (24%) survived past the first month of life and 48 (76%) did not.

The preterm delivery rate among women with known pregnancy outcomes was 13.6% compared with 9.7% (3704/38 189) in similar women in 2020, a 40% increase.^17^

### Health service use

In the 2019 to 2020 multiple indicator cluster survey,^4^ 99.4% of deliveries in the Gaza Strip were facility-based, with government hospitals handling 78.3% (Table 2). After the start of the conflict, the proportion of facility-based deliveries remained high among women with known pregnancy outcomes, at 97.4% (12 531/12 868), but the proportion of deliveries in government hospitals was lower, at 60.8% (7828/12 868). For other locations, the proportions were 31.0% (3990/12 868) for nongovernmental organization hospitals, 3.6% (466/12 868) for private sector facilities, 1.9% (247/12 868) and 0.1% (11/12 868) for field hospitals and medical points (which did not exist previously), respectively, and 1.7% (220/12 868) for homes, shelters, tents and ambulances.

**Table 2:** Health service use, study of maternal and neonatal outcomes in pregnant women, Gaza Strip, 2023–2024

Health service use indicator	Indicator value			
	Weighted %^a^		No. of women (%; 95% CI)^b^	
	2019–2020 live births among non-refugees and UNRWA refugees^c^ (*n* = 1009)		UNRWA 2020 e-health records of live births and stillbirths among refugees (*n* = 38 189)	Live births and stillbirths to refugee women recorded by UNRWA as pregnant on 8 October^d^ (*n* = 12 868)
**Location of delivery**				
All health-care facilities	99.4		38 172 (99.9; 99.9–100)	12 531 (97.4; 97.1–97.7)
Government hospital	78.3		38 014 (99.5; 99.5–99.6)	7 828 (60.8; 60.0–61.7)
NGO hospital	5.1		0 (0; NA)	3 990 (31.0; 30.2–31.8)
Private hospital	15.7		158 (0.4; 0.3–0.5)	466 (3.6; 3.3–3.9)
Field hospital	0.0		0 (0; NA)	247 (1.9; 1.7–2.2)
Medical point	0.0		0 (0; NA)	11 (0.1; 0.0–0.1)
Home, shelter, tent or ambulance	0.4		17 (0.04; 0.0–0.1)	220 (1.7; 1.5–1.9)
Missing data	NA		0.0 (0; NA)	106 (0.8; 0.7–1.0)
**Birth attendant**				
All skilled birth attendants	100.0		ND	12 534 (97.4; 97.1–97.7)
Medical doctor	87.5		ND	9 818 (76.3; 75.6–77.0)
Nurse or midwife	12.5		ND	2 716 (21.1; 20.4–21.8)
Relative, friend, family member or daya	0.0		ND	125 (1.0; 0.8–1.1)
No attendant	0.0		ND	39 (0.3; 0.2–0.4)
Missing data	NA		ND	170 (1.3; 1.1–1.5)
**Caesarean section rate**				
All health-care facilities	22.5		9 770 (25.6; 25.1–26.0)	2 512 (19.5; 18.8–20.2)
Government hospitals	22.5		1 366 (17.5; 16.6–18.3)^f^	1 366 (17.5; 16.6–18.3)^f^
NGO hospitals	16.0		990 (24.8; 23.5–26.2)^f^	990 (24.8; 23.5–26.2)^f^
Private hospitals	34.5		97 (20.8; 17.1–24.5)^f^	97 (20.8; 17.1–24.5)^f^
Field hospitals	NA		53 (21.5; 16.3–26.6)^f^	53 (21.5; 16.3–26.6)^f^
**Length of facility stay in hours, median (IQR)**				
All births	36 (6–60)		ND	6 (3–14)^g^
Vaginal deliveries	20 (6–36)		ND	6 (3–10)^g^
Caesarean section deliveries	60 (36–84)		ND	24 (12–24)^g^
**Women staying in facility < 24 h after vaginal delivery**	58.6		ND	5 221 (88.9; 88.1–89.7)^h^
**Women staying in facility < 72 h after caesarean section delivery**	72.8		ND	1 427 (93.9; 92.7–95.1)^h^

CI: confidence interval; IQR: interquartile range; NA: not applicable; ND: not determined; NGO: nongovernmental organization: UNRWA: United Nations Relief and Works Agency for Palestine Refugees in the Near East.

^a^ The numbers listed are percentages, unless otherwise indicated.

^b^ The figures listed are the number of women, percentages and 95% confidence intervals, unless otherwise indicated.

^c^ Numbers for individual indicators were not available from the multiple indicator cluster survey as sampling weighting had been applied.^4^

^d^ Data on pregnancy outcome were obtained by phone survey and from e-health records established by UNRWA.

^e^ A *daya* is a traditional birth attendant.

^f^ The denominator was the number of births that took place in the specific location.

^g^ This information was available for only 57.4% of the sample (i.e. for 7394 women who gave birth).

^h^ The denominator was the number of women who gave birth by that specific mode of delivery (i.e. vaginal or caesarean section).

Among women with known pregnancy outcomes, the presence of a skilled birth attendant remained high, at 97.4% (12 534/12 868) compared with 100% in 2019 to 2020 (Table 2),^4^ but the composition of the providers changed. The proportion of births attended by a medical doctor decreased from 87.5% to 76.3% (9818/12 868), whereas the proportion attended by a nurse or midwife increased from 12.5% to 21.1% (2716/12 868). After the start of the conflict, the proportion attended by a relative or friend was 1.0% (125/12 868) and the proportion of unattended births was 0.3% (39/12 868). The caesarean section rate decreased somewhat from 25.6% among UNRWA refugees in 2020 to 19.5% (2512/12 868) among women with known pregnancy outcomes in our study.

The median length of stay in a facility after delivery was shorter among women with known pregnancy outcomes than among the Gazan population pre-conflict. For vaginal deliveries, the median stay was 20 h (inter-quartile range, IQR: 6–36) in 2019 to 2020 and 6 h (IQR 3–10) in our study (Table 2).^4^ For caesarean section births, the median stay was 60 h (IQR: 36–84) in 2019 to 2020 and 24 h (IQR 12–24) in our study. Correspondingly, the proportion of women discharged within 24 h of vaginal delivery increased from 58.6% in 2019 to 2020 to 88.9% (5221/5874), and the proportion discharged within 72 h of caesarean section increased from 72.8% in 2019 to 2020 to 93.9% (1427/1520).

## Discussion

This study, which collected data from Palestinian Ministry of Health lists of deaths, UNRWA e-health records and an UNRWA phone survey, documents the serious deterioration in maternal and neonatal health among refugees in the Gaza Strip following the onset of the conflict on 7 October 2023. Moreover, roughly a quarter of women in our study cohort were unreachable by phone. As these women and their babies probably had the worst outcomes and the worst experiences of using health care, our findings are likely to represent a best-case scenario and to have underestimated adverse events.

Our analysis revealed that pregnancy-related, stillbirth and neonatal mortality in the Gaza Strip in late 2023 and 2024 were higher than in previous years. In 2022, 13 (non-trauma related) maternal deaths were reported in the Gaza Strip among refugee and non-refugee populations combined, corresponding to a maternal mortality ratio of 17 per 100 000 live births.^1^^,^^20^ In our study cohort, the pregnancy-related mortality ratio was 948 per 100 000 pregnancies after the start of the conflict. Once the underreporting of deaths by the Palestinian Ministry of Health is considered, the pregnancy-related mortality ratio may have been 1468 per 100 000 pregnancies. Overall, almost half of deaths identified through our phone survey were not on health ministry lists, and a previous study indicated that these lists underreported all deaths by 41% (95% CI: 32–52).^18^ In our results, we present a wide range for the pregnancy-related mortality ratio, from a lower bound based on observed deaths among women with known pregnancy outcomes to an upper bound derived by incorporating the underreporting of deaths among women with unknown pregnancy outcomes, which gave a plausible uncertainty range between 390 and 4723 deaths per 100 000 pregnancies. Nevertheless, the uncorrected pregnancy-related mortality ratio of 948 per 100 000 deaths among all pregnant women represents a more than 30-fold increase over the average maternal or pregnancy-related mortality ratio in the Gaza Strip for 2018 to 2022 (i.e. 30 per 100 000 live births) and exceeds the increases documented in other conflicts.^1^^,^^23^

We recorded pregnancy-related mortality rather than maternal mortality because it was not feasible to conduct verbal autopsies by phone under conflict conditions. In fact, the UNRWA social autopsy investigation also faced substantial challenges, including difficulty contacting women due to phone or network problems, siege conditions and attacks on hospitals and missing data (such as dates of death), which made it hard to establish an accurate denominator for the total number of women giving birth.^22^ The widely used time-of-death definition does not require a cause of death to be established. In hindsight, it would have been useful to ascertain, via a single question, if a death was traumatic. Nevertheless, the relatively small number of maternal deaths recorded in the social autopsy compared to the number of pregnancy-related deaths we identified suggests that the main cause of death among pregnant women and women who had recently given birth in the Gaza Strip was probably trauma. This interpretation is reinforced by the absence of a J-shaped pattern for age-specific mortality ratios.^24^

When mothers died, their babies’ outcomes were dire. Among live births to mothers who died, the neonatal mortality rate was astronomical, at 444 per 1000 live births, which was 47 times higher than among live births to mothers who survived. This excess was higher than that estimated in a systematic review of low- and middle-income countries, which found that the risk of death among neonates whose mothers died was 11.3 times that among neonates whose mother survived.^25^ We also observed elevated stillbirth and neonatal mortality rates, which aligns with existing research on conflicts.^23^

The conflict also precipitated a shift in delivery location, probably due to extensive damage to the health infrastructure by the Israeli armed forces.^9^^–^^11^ Deliveries in government hospitals decreased, whereas those in nongovernmental organization hospitals and field hospitals increased. Concurrently, the median length of post-delivery stay in health facilities decreased sharply, possibly because of shortages in nursing staff and the precarious situation in health facilities.^12^ This reduction, combined with more deliveries in field hospitals and at home, may have influenced the quality of care and increased the risk of maternal and neonatal complications. These concerns are further compounded by the increase in the preterm birth rate we observed, which may have been linked to conflict-induced stress, widespread malnutrition and absent or inadequate antenatal care services.^26^ Our findings reflect service use among women with known pregnancy outcomes; service use may have been less and the preterm birth rate may have been higher among women with unknown pregnancy outcomes.

Our study’s strengths are the quantity of information available on women who were pregnant on 8 October 2023 and our use of multiple, linked data sources, which provided a comprehensive and nuanced view of the situation in the Gaza Strip. This triangulation approach was critical because the phone survey identified only 67 pregnancy-related deaths, whereas combining data from the phone survey and Palestinian Ministry of Health lists revealed between 166 and 171 deaths, depending on when the deaths were assumed to have occurred in each month. Our approach underscores the importance of using multiple data sources to accurately assess outcomes in complex emergencies.

Implementing effective maternal and perinatal death surveillance and response systems during humanitarian crises or conflicts is challenging. The identification, reporting and review processes around deaths can be severely compromised.^27^ The underreporting of deaths we observed aligns with experiences in other conflict zones where mortality surveillance is hindered by security concerns, damaged infrastructure and overwhelmed health systems. Nevertheless, the multisource approach to data collection we employed is a pragmatic way of meeting these challenges. Our study was greatly aided by UNRWA's antenatal care records of pregnancies which could be followed up to ascertain outcomes. However, our reliance on UNRWA records also meant that our findings primarily reflected the experience of the refugee population in the Gaza Strip and their generalizability to the entire population may be limited. We recognize that the risk of death and access to health care may have been different for unregistered refugees and non-refugee Palestinians.

Our study had some limitations. First, we used Palestinian ID numbers as unique identifiers to link UNRWA data and Palestinian Ministry of Health lists of deaths. However, mismatches could have occurred because of data entry errors in either system, as well as because of underreporting of deaths by the Palestinian Ministry of Health. Consequently, we may have underestimated mortality rates if deaths were missed or, more improbably, overestimated rates if there were false-positive links. Not all women in the study cohort had completed the 42-day period after their expected delivery dates or had given birth, which could also have led to an underestimate of the risk of death. Second, recall bias may have affected phone survey responses, particularly regarding the exact timing of events. Although our use of pregnancy-related mortality rather than maternal mortality affected comparability with estimates from before the conflict, it was necessary given data collection constraints during active conflict. Third, stillbirth and neonatal mortality rates among women with unknown pregnancy outcomes were estimated from rates among women with known pregnancy outcomes by assuming a ratio derived from the Palestinian Ministry of Health underreporting of pregnancy-related deaths among women with known pregnancy outcomes.

Our study is an attempt to systematically document maternal and perinatal mortality during an active conflict. The study provides evidence that the conflict in the Gaza Strip severely affected maternal health outcomes and patterns of access to health care. These findings demand an immediate and sustained international response focused on: (i) supporting a ceasefire to end traumatic deaths due to bombing; (ii) protecting health-care facilities and personnel; (iii) ensuring unimpeded access to emergency obstetric and newborn care; (iv) addressing the underlying determinants of maternal and neonatal ill-health, including problems with food security, water and sanitation; and (v) supporting families in which a mother who died had given birth to a live infant. In the longer term, we would also argue for: investing in rebuilding an equitable health system for the Gaza Strip and conducting a comprehensive needs assessment.
