# Corrigendum

**DOI:** 10.2471/BLT.26.101126

**Published:** 2026-11-01

In: Jamaluddine Z, Jaffal R, Al Najjar S, Al Kahlout M, Abou Zeid T, et al. Maternal and neonatal outcomes in a cohort of pregnant women during conflict, Gaza Strip. Bull World Health Organ. 2026 Jun 1;104(12):386–95, 

on page 390, the last sentence on the first column should read as follows:

"The ratio of the estimated number of pregnancy-related deaths number of pregnancy-related deaths after correction for Palestinian Ministry of Health underreporting (i.e. 193) to the number of pregnancy-related deaths among women with known pregnancy outcomes (i.e. 67) was 2.88."

